# Supplementary material for: Inflammatory and Cardiac Biomarkers in Relation with Post-Acute COVID-19 and Mortality: What We Know after Successive Pandemic Waves
Source: Diagnostics (Basel). 2022 Jun 2;12(6):1373. doi: 10.3390/diagnostics12061373 (PMC9222082; doi:10.3390/diagnostics12061373)
Supplement: Supplementary file 1 [file diagnostics-12-01373-s001.zip › diagnostics-1726111-supplementary.pdf]

diagnostics

MDPI

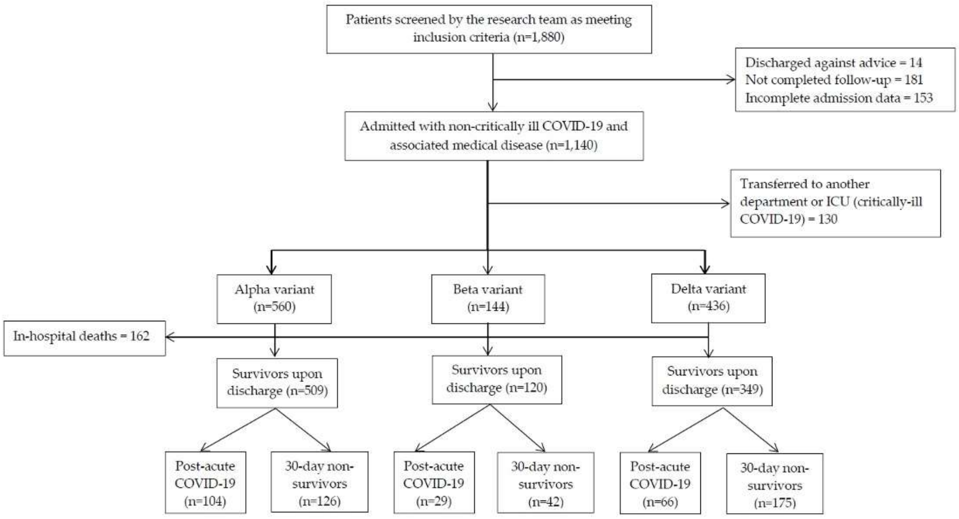

Figure S1. Flowchart of the study.

Table S1. Correlations between inflammation biomarkers and the type of symptoms of post-acute COVID-19 recorded.

Biomarker	Symptom category	Value	Symptom category	Value	*p*-value*
hs-CRP (mg/dL)	Respiratory	12.67±13.72	Systemic	7.54±7.56	0.012
SII	Neurocognitive	3646±2467	Systemic	1767±2161	0.024
			Musculo-skeletal	1217±807	0.008
			Cardiovascular	1584±1374	0.015
			Psychological&social	1473±1076	0.015
			Respiratory	1755±1960	0.030
MLR	Musculo-skeletal	1.13±1.56	Systemic	0.61±0.42	0.001
			Cardiovascular	0.56±0.24	<0.001
			Psychological&social	0.51±0.20	0.001
			Neurocognitive	0.53±0.18	0.042
			Respiratory	0.54±0.42	0.001
RDW (%)	Psychological&social	15.42±3.84	Systemic	13.80±1.83	0.004
			Musculo-skeletal	13.47±1.24	0.010
			Cardiovascular	14.07±2.93	0.025
WBC (*1,000/microL)	Systemic	8.41±4.44	Respiratory	20.75±7.24	0.044

Data are reported as mean±SD; *multiple comparisons with Anova; hs-CRP, high sensitivity C-reactive protein; SII, systemic inflammatory index; MLR, monocyte-to-lymphocyte ration; RDW, red cell distribution width (%, coefficient of variation); WBC, white blood cells.

Table S2. Univariate and multivariate significant predictors for 30-days mortality in non-critically ill COVID-19 patients.

SARS-CoV-2 variant	Variable	Univariate analysis		Multivariate analysis	
		HR (95% CI)	*p*-Value	HR (95% CI)	*p*-Value
Alpha Variant	Age	1.05 (1.02-1.07)	<0.001	1.11 (1.02-1.20)	0.017
	CCI	1.42 (1.27-1.58)	<0.001		
	NEWS 2 score	1.50 (1.34-1.67)	<0.001	1.66 (1.09-2.55)	0.020
	History of AD	1.84 (1.06-3.19)	0.030		
	History of AF	0.32 (0.19-0.52)	<0.001		
	Respiratory rate	1.09 (1.02-1.17)	0.014		
	Discharge CRP	1.08 (1.03-1.13)	<0.001		

	Discharge hsTnI	1.87 (1.24-2.81)	0.003		
	NT-proBNP	3.74 (2.43-5.75)	<0.001	4.65 (1.05-20.62)	0.043
	ACS	0.26 (0.10-0.70)	0.008		
	AHF	2.69 (1.58-4.56)	<0.001		
	Oxygen therapy	0.15 (0.08-0.29)	<0.001		
	Hospitalization (days)	0.93 (0.89-0.96)	<0.001		
Beta Variant	Age	1.04 (1.01-1.08)	0.024		
	NEWS 2 score	1.22 (1.06-1.41)	0.005		
	Respiratory rate	1.13 (1.01-1.27)	0.033		
	Discharge CRP	1.07 (1.00-1.15)	0.056		
	Discharge hsTnI	3.29 (0.81-13.32)	0.095		
	NT-proBNP	2.28 (1.22-4.26)	0.010	2.03 (0.99-4.16)	0.053
	Oxygen therapy	0.18 (0.04-0.80)	0.024		
Delta Variant	Age	1.02 (1.00-1.04)	0.034		
	Discharge hsTnI	4.01 (0.79-20.44)	0.095		
	NT-proBNP	2.23 (1.27-3.91)	0.005	3.24 (0.95-11.06)	0.061
	Oxygen therapy	0.17 (0.04-0.72)	0.016		
	Hospitalization (days)	0.88 (0.83-0.93)	<0.001	0.78 (0.68-0.90)	0.001

AD, atherosclerotic disease; AF, atrial fibrillation; CRP, C-reactive protein; hs-TnI, high-sensitivity TnI; NT-proBNP, N-terminal pro-B-type natriuretic peptide; ACS, acute coronary syndrome; AHF, acute heart failure; NEWS, National Early Warning Score.

Table S3. Biomarkers associated with 30-days mortality.

Factors	HR (95%CI)	P value
**Univariate analysis**		
NT-proBNP	1.74 (0.19-2.76)	0.019
hs-TnI	0.88 (0.62-1.94)	0.464
MLR	0.95 (0.74-1.21)	0.661
RDW	1.02 (0.96-1.09)	0.491
NLR	1.02 (1.01-1.03)	0.038
Virus variant (Delta)	0.48 (0.28-0.83)	0.009
**Multivariate analysis**		
NT-proBNP	1.86 (1.00-3.47)	0.052
hs-TnI	0.79 (0.50-1.23)	0.294
MLR	0.64 (0.38-1.06)	0.084
RDW	1.05 (0.96-1.15)	0.260
NLR	1.06 (1.01-1.11)	0.015
Virus variant (Delta)	0.48 (0.23-0.98)	0.043

HR, hazard ratio; CI, confidence interval; NT-pro BNP, N-terminal B-type natriuretic peptide; hs-TnI, high sensitivity cardiac troponin I; MLR, monocyte-to-lymphocyte ratio; RDW, red cell distribution width; NLR, neutrophil-to-lymphocyte ratio.

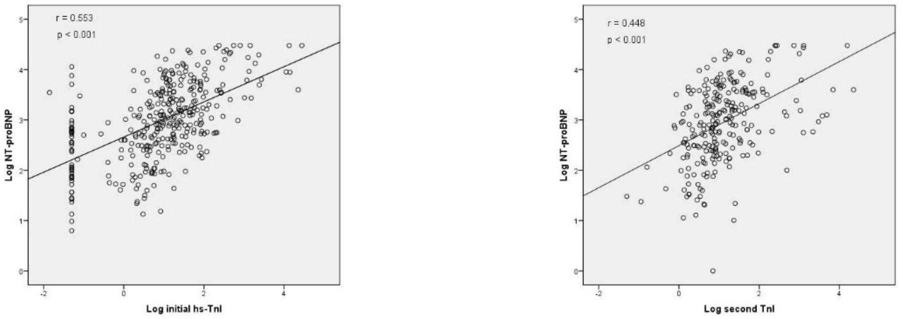

(**a**) (**b**)

Figure S2. (**a**) Dot plot demonstrating a moderate correlation between the logarithmic concentrations of NT-pro BNP and the initial hs-TnI values; (**b**) Dot plot demonstrating a moderate correlation between the logarithmic concentrations of NT-pro BNP and the predischarge hs-TnI assessment.

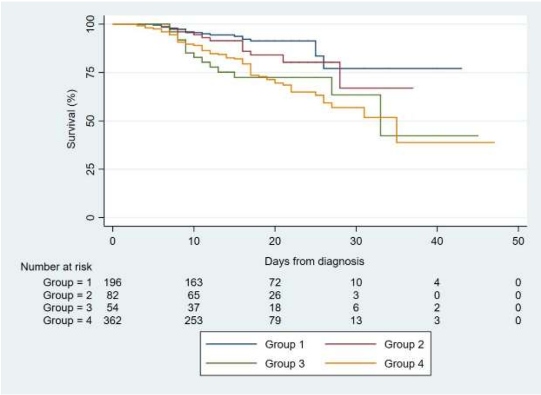

Figure S3. Kaplan–Meier survival curves regarding all-cause mortality according to the groups of NT-pro BNP excluding those patients who developed acute heart failure after SARS-CoV-2 infection.

Table S4. Complementary analysis for Cox-proportional hazards model assessing the relationship between NT-proBNP and mortality during follow-up adjusted for relevant covariates, including hs-TnI.

Variable	Univariable			Multivariable		
	HR (95%CI)	SE	P value	HR (95%CI)	SE	P value
hs-TnI (per log. Unit)	1.71(1.21-2.42)	0.18	0.002	1.55(1.0-2.42)	0.23	0.051
NT-proBNP (per log. Unit)	1.96 (1.54-2.50)	0.12	<0.001	1.29(1.07-1.56)	0.10	0.007
Age (per 10 years)	1.03 (1.02-1.04)	0.01	<0.001	1.03(1.01-1.05)	0.01	0.007
Male sex	0.82 (0.61-1.08)	0.15	0.159	0.88(0.58-1.32)	0.21	0.525
NEWS2 score	1.16 (1.11-1.21)	0.02	<0.001	1.14(1.04- 1.24)	0.04	0.004
Admission SaO2 <90%	0.46 (0.35-0.61)	0.15	<0.001	1.15(0.65-2.02)	0.29	0.635
History of AD	1.03 (0.71-1.49)	0.19	0.895	0.89(0.54-1.48)	0.26	0.658
History of HT	0.87 (0.61-1.25)	0.18	0.449	1.26(0.83-1.90)	0.21	0.280
History of DM	1.78 (1.27-2.49)	0.17	0.001	1.00(0.31-3.25)	0.60	0.997
History of CKD	0.39(0.24-0.64)	0.25	<0.001	0.59(0.32-1.12)	0.32	0.105
History of HF	0.72 (0.49-1.04)	0.19	0.082	1.89(1.01-3.56)	0.32	0.047
SBP (per 10 mmHg)	0.99 (0.98-1.01)	0.01	0.089	0.49(0.05-4.69)	1.15	0.535
AHF on admission	0.81 (0.57-1.15)	0.18	0.240	0.79(0.40-1.53)	0.34	0.476
ACS on admission	2.22(1.17-4.21)	0.33	0.015	0.49(0.23-1.05)	0.39	0.066
Mechanical ventilation	0.84 (0.50-1.41)	0.27	0.500	1.11(0.61-2.02)	0.31	0.731
Corticotherapy	0.63 (0.45-0.87)	0.17	0.006	1.35(0.91-2.01)	0.20	0.133
4th pandemic wave (Delta variant)	2.01 (1.66-2.43)	0.10	<0.001	1.21(0.92-1.59)	0.14	0.175
CCI	1.16 (1.08-1.23)	0.03	<0.001	1.09(0.98-1.21)	0.05	0.105

HR, hazard ratio; CI, confidence interval; SE, standard error; hs-TnI, high-sensitivity troponin I; NT-proBNP, N-terminal pro B-type natriuretic peptide; NEWS2, National Early Warning Score 2; SaO_2, oxygen saturation; AD, atherosclerotic disease; HT, arterial hypertension; DM, diabetes mellitus; CKD, chronic kidney disease; HF, chronic heart failure; SBP, systolic blood pressure; AHF, acute heart failure; CCI, Charlson comorbidity index.

Table S5. Complementary analysis for Cox-proportional hazards model assessing the relationship between NT-proBNP and mortality during follow-up adjusted for relevant covariates, including D-dimer.

Variable	Univariable			Multivariable		
	HR (95% CI)	SE	P value	HR (95% CI)	SE	P value
NT-proBNP (per log. unit)	1.96 (1.54-2.50)	0.12	<0.001	1.36 (0.99-1.89)	0.17	0.060
Age (per 10 years)	1.03 (1.02-1.04)	0.01	<0.001	24.12 (0.84-693.56)	1.71	0.063
Male sex	0.82 (0.61-1.08)	0.15	0.159	0.85 (0.55-1.32)	0.22	0.476
NEWS2 score	1.16 (1.11-1.21)	0.02	<0.001	1.09 (0.99-1.21)	0.05	0.096
Admission SaO_2 <90%	0.46 (0.35-0.61)	0.15	<0.001	0.89 (0.48-1.66)	0.32	0.720
History of AD	1.03 (0.71-1.49)	0.19	0.895	0.94 (0.56-1.58)	0.27	0.817
History of HT	0.87 (0.61-1.25)	0.18	0.449	1.12 (0.72-1.74)	0.22	0.607
History of DM	1.78 (1.27-2.49)	0.17	0.001	1.19 (0.37-3.90)	0.60	0.768
History of CKD	0.39(0.24-0.64)	0.25	<0.001	0.60 (0.32-1.16)	0.33	0.127
History of HF	0.72 (0.49-1.04)	0.19	0.082	1.56 (0.81-3.02)	0.34	0.185
SBP (per 10 mmHg)	0.99 (0.98-1.01)	0.01	0.089	0.22 (0.02-3.01)	1.33	0.257
AHF on admission	0.81 (0.57-1.15)	0.18	0.240	0.74 (0.37-1.50)	0.36	0.411
Mechanical ventilation	0.84 (0.50-1.41)	0.27	0.500	0.33 (0.15-0.71)	0.39	0.005
Corticotherapy	0.63 (0.45-0.87)	0.17	0.006	1.36 (0.90-2.05)	0.21	0.145
4th pandemic wave (Delta)	2.01 (1.66-2.43)	0.10	<0.001	0.94 (0.47-1.87)	0.35	0.858
CCI	1.16 (1.08-1.23)	0.03	<0.001	1.13 (1.01-1.26)	0.06	0.029
D-dimer (per log. unit)	1.88 (1.08-3.28)	0.29	0.027	1.01 (0.99-1.04)	0.01	0.309

HR, hazard ratio; CI, confidence interval; SE, standard error; NT-proBNP, N-terminal pro B-type natriuretic peptide; NEWS2, National Early Warning Score 2; SaO_2, oxygen saturation; AD, atherosclerotic disease; HT, arterial hypertension; DM, diabetes mellitus; CKD, chronic kidney disease; HF, chronic heart failure; SBP, systolic blood pressure; AHF, acute heart failure; CCI, Charlson comorbidity index.

Table S6. Biomarkers associated with post-acute COVID-19.

Factors	HR (95%CI)	P value
**Univariate analysis**		
NT-proBNP	1.70 (1.09-2.66)	0.020
hs-TnI*	0.47 (0.34-0.63)	< 0.001
MLR	2.06 (0.98-4.36)	0.058
RDW	0.86 (0.74-0.99)	0.041
Virus variant (Delta)	1.21 (0.48-3.05)	0.044
**Multivariate analysis**		
NT-proBNP	1.68 (1.00-2.84)	0.05
hs-TnI*	0.44 (0.31-0.62)	< 0.001
MLR	2.77 (1.10-6.94)	0.03
RDW	0.91 (0.77-1.07)	0.233
Virus variant (Delta)	0.68 (0.24-1.91)	0.465

HR, hazard ratio; CI, confidence interval; NT-pro BNP, N-terminal B-type natriuretic peptide; hs-TnI, high sensitivity cardiac troponin I; *, values obtained predischarge; MLR, monocyte-to-lymphocyte ratio; RDW, red cell distribution width; NLR, neutrophil-to-lymphocyte ratio.

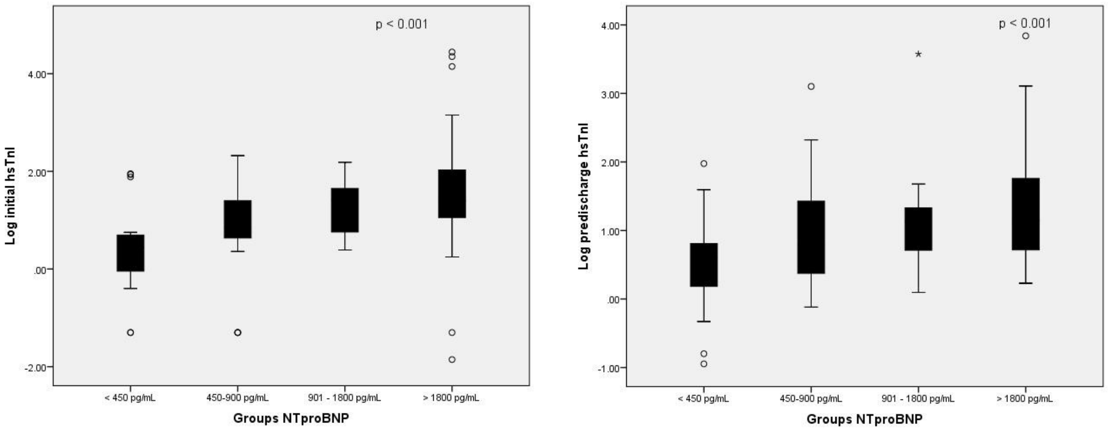

(**a**) (**b**)

Figure S4. (**a**) Boxplot demonstrating the logarithmic concentrations of initial hs-TnI among the NT-pro BNP groups; (**b**) Boxplot demonstrating the logarithmic concentrations of predischarge hs-TnI among the NT-pro BNP groups.
